# Navigating AI transitions: how coaching leadership buffers against job stress and protects employee physical health

**DOI:** 10.3389/fpubh.2024.1343932

**Published:** 2024-03-27

**Authors:** Jeeyoon Jeong, Byung-Jik Kim, Julak Lee

**Affiliations:** ^1^Business School, Korea University, Seoul, Republic of Korea; ^2^College of Business, University of Ulsan, Ulsan, Republic of Korea; ^3^Department of Psychology, Yonsei University, Seoul, Republic of Korea; ^4^Department of Industrial Security, Chung-Ang University, Seoul, Republic of Korea

**Keywords:** artificial intelligence adoption, job stress, physical health, coaching leadership, moderated mediation model

## Abstract

The dynamic interplay between Artificial Intelligence (AI) adoption in modern organizations and its implications for employee well-being presents a paramount area of academic exploration. Within the context of rapid technological advancements, AI’s promise to revolutionize operational efficiency juxtaposes challenges relating to job stress and employee health. This study explores the nuanced effects of Artificial Intelligence (AI) adoption on employee physical health within organizational settings, investigating the potential mediating role of job stress and the moderating influence of coaching leadership. Drawing from the conservation of resource theory, the research hypothesized that AI adoption would negatively impact employee physical health both directly and indirectly through increased job stress. Critically, our conceptual model underscores the mediating role of job stress between AI adoption and physical health. Further, introducing a novel dimension to this discourse, we postulate the moderating influence of coaching leadership. To empirically test the hypotheses, we gathered survey data from 375 South Korean workers with a three-wave time-lagged research design. Our results demonstrated that all the hypotheses were supported. The results have significant implications for organizational strategies concerning AI implementation and leadership development.

## Introduction

In the current organizational landscape characterized by rapid technological transformations and emerging trends, the relationship between technological adoption and employee well-being has gained significant academic interest (1). This evolving context places an increased emphasis on the importance of employees’ physical health, moving it from a secondary consideration to a primary element of organizational performance (2, 3). Indeed, the well-being of employees is now recognized as a central determinant of organizational outcomes, closely tied to factors such as productivity, innovation, resilience, and the broader organizational culture (4, 5). Artificial Intelligence (AI) is positioned at the heart of this context, offering both promising advancements and associated challenges (6–8).

Artificial Intelligence (AI), as understood in the current organizational context, encompasses a range of technologies designed to replicate human cognitive processes, thus enabling enhanced problem-solving capabilities and task execution (9). Specifically, AI is defined as a suite of interconnected technologies geared toward performing tasks or solving problems that, when conducted by humans, necessitate cognitive processes (10). This includes capabilities such as machine learning algorithms for pattern recognition in vast datasets, natural language processing tools for text extraction and classification, and decision support frameworks that augment human decision-making abilities (11). Most previous research on AI has been focused on its potential benefits or bright side, such as optimizing operations, enhancing productivity, fostering innovation, and facilitating decision-making processes (7–9, 12–15). Yet, there is a relative paucity of studies examining the potential adverse or dark side effects of AI on employees, such as increased job stress, feelings of obsolescence, or concerns about job security (16–19).

As AI continues its upward trajectory, it not only signifies a monumental shift in operational efficiency but also presents intricate challenges and ambiguities concerning its impact on human resources (20). The incorporation of AI, often characterized as a pivotal facet of the fourth industrial revolution, underscores the need for a comprehensive exploration of its overarching effects on the workforce (10). While current scholarship predominantly underscores AI’s potential to revolutionize job roles, alter organizational structures, and effectuate sectoral transformations, there is an emergent yet underrepresented discourse addressing the potential amplification of job-related stress and its subsequent implications for employee well-being, both mental (e.g., job stress) and physical health (19, 21).

From the vast repository of existing literature, existing literature reveals notable research gaps. Primarily, the intricate relationship between AI adoption and its effects on employees’ physical health requires further exploration (9). As physical health becomes a central focus within organizations, it is crucial to understand the potential impacts of technological advancements in this domain (4). Moreover, the overarching framework connecting AI adoption to job stress and subsequent health outcomes remains in its formative stages. A deeper exploration is essential, especially regarding job stress as a mediator, to fully grasp the dynamics between AI and overall well-being (22). Organizations on the path of AI-centric transformation will find such insights invaluable.

Additionally, the role of leadership, and in particular, coaching leadership, in the relationship between AI and employees’ mental health (e.g., job stress) is yet to be thoroughly understood. Leadership plays a pivotal role in shaping the organizational environment (23), and its potential to buffer the challenges posed by AI is significant. Coaching leadership, with its focus on education, mentoring, and employee development, could potentially mitigate the stresses stemming from AI transitions, thereby promoting employee health (24, 25). This manuscript aims to address these gaps by delving deeply into the relationship between AI and physical health, taking into account the mediating influence of job stress and the moderating role of coaching leadership. To explain this, we apply the principles of the conservation of resource theory (COR theory) (26). Based on the COR theory’s key idea that individuals aim to obtain, maintain, and safeguard their resources, we suggest that the adoption of AI in organizations might act as a challenge, potentially draining these resources (27). Such technological shifts can place increased demands on employees, escalating their stress levels, which could lead to a decline in physical health (28).

However, we argue that coaching leadership can compensate for the negative effect of AI adoption on employees’ job stress as it acts as employees’ necessary resources to handle the job stress in response to AI adoption. The COR theory further suggests ways to gather and build resources that can counter these challenges (29). Within our framework, we see coaching leadership as a critical resource for organizations. Strong coaching leadership can offer the necessary support, guidance, and strategies that help employees navigate the challenges brought by AI (25, 30–32). When organizations emphasize such leadership, it can act as a protective shield, reducing the stress associated with AI changes (33). In turn, this promotes better physical health among employees. In contrast, without this leadership style, the negative effects of AI could amplify, leading to increased stress and deteriorating health.

In sum, our model presents a layered relationship where AI adoption might affect physical health, with job stress serving as a bridge and coaching leadership acting as a pivotal influencer. Drawing from the COR theory, this framework offers a clearer understanding of how AI, job stress, and leadership intertwine to impact employee health (34). This approach not only addresses the identified research gaps but also sets the stage for future investigations in the field.

This academic paper makes four notable contributions to the existing body of knowledge on the intersection of technology and organizational well-being. First, this paper significantly advances our understanding of the indirect effects of AI on employee physical health by establishing job stress as a full mediator. This contribution is particularly important as it moves beyond the traditional focus on direct effects and instead highlights the psychological processes that link technological change to physical well-being. It thereby responds to calls within the literature for more nuanced models that consider the intermediary variables that might influence the relationship between AI adoption and health outcomes. Second, by applying the COR theory to the context of AI in the workplace, the paper extends this theoretical framework in a novel direction. The study demonstrates how the theory can be used to predict not only psychological outcomes but also physical health impacts related to technology adoption. This theoretical contribution provides a new lens for understanding how the introduction of AI in the workplace threatens employees’ resources and thereby catalyzes stress responses with physiological consequences. Third, the paper identifies coaching leadership as a crucial moderating variable that can alleviate the stress employees may experience due to AI adoption. This finding contributes to leadership literature by emphasizing the importance of coaching behaviors in technological transitions. It also offers a practical contribution by suggesting that organizations should invest in leadership development as a strategy to protect employee well-being during periods of technological upheaval. Lastly, through its methodological rigor, particularly the use of three-wave time lagged data; the paper sets a high standard for empirical research in the field. The robust sample size and statistical techniques employed also ensure the reliability and validity of the findings, serving as a methodological blueprint for future studies investigating complex models of technology’s impact on employees.

In addition, in the introduction section of an academic paper focusing on the impacts of Artificial Intelligence (AI) adoption within organizations, it is imperative to delineate clearly the conceptual underpinnings of both physical health and mental health, particularly in the context of job stress. This clarity is essential not only for establishing the scope of the study but also for ensuring that readers understand the specific domains of employee well-being being investigated. Physical health, in the context of this research, should be defined as the condition of an individual’s body, encompassing the absence of physical disease, physical injury, and the presence of physical fitness. It involves various physiological metrics including, but not limited to, cardiovascular health, metabolic health indicators, musculoskeletal condition, and overall energy levels (19, 21). The operationalization of physical health in the study must be explicit, detailing the specific physical outcomes being examined, whether they are self-reported health statuses, clinical health metrics, or physical symptoms related to workplace stress (19). And, mental health, with a specific focus on job stress within this paper, refers to the psychological and emotional state that affects an individual’s ability to perform effectively in their work environment. Job stress is conceptualized as the response to work conditions that challenge or exceed an individual’s resources and coping abilities. This can include, but is not limited to, factors such as role ambiguity, workload, job insecurity, and lack of control over job processes (35, 36). By explicitly defining physical and mental health in the introduction, the authors can set the stage for a nuanced exploration of the interplay between these aspects of well-being in the context of AI adoption. This approach not only enhances the conceptual clarity of the study but also aligns with academic rigor by situating the research within the broader discourse on occupational health psychology. Furthermore, detailing these definitions helps in establishing a theoretical framework that guides the study’s hypotheses, methodology, and interpretation of findings, ensuring that the research contributes meaningfully to the existing body of knowledge on the impacts of technological advancements on employee health.

## Theory and hypotheses

### The impact of AI adoption on employee physical health

**Figure 1:**
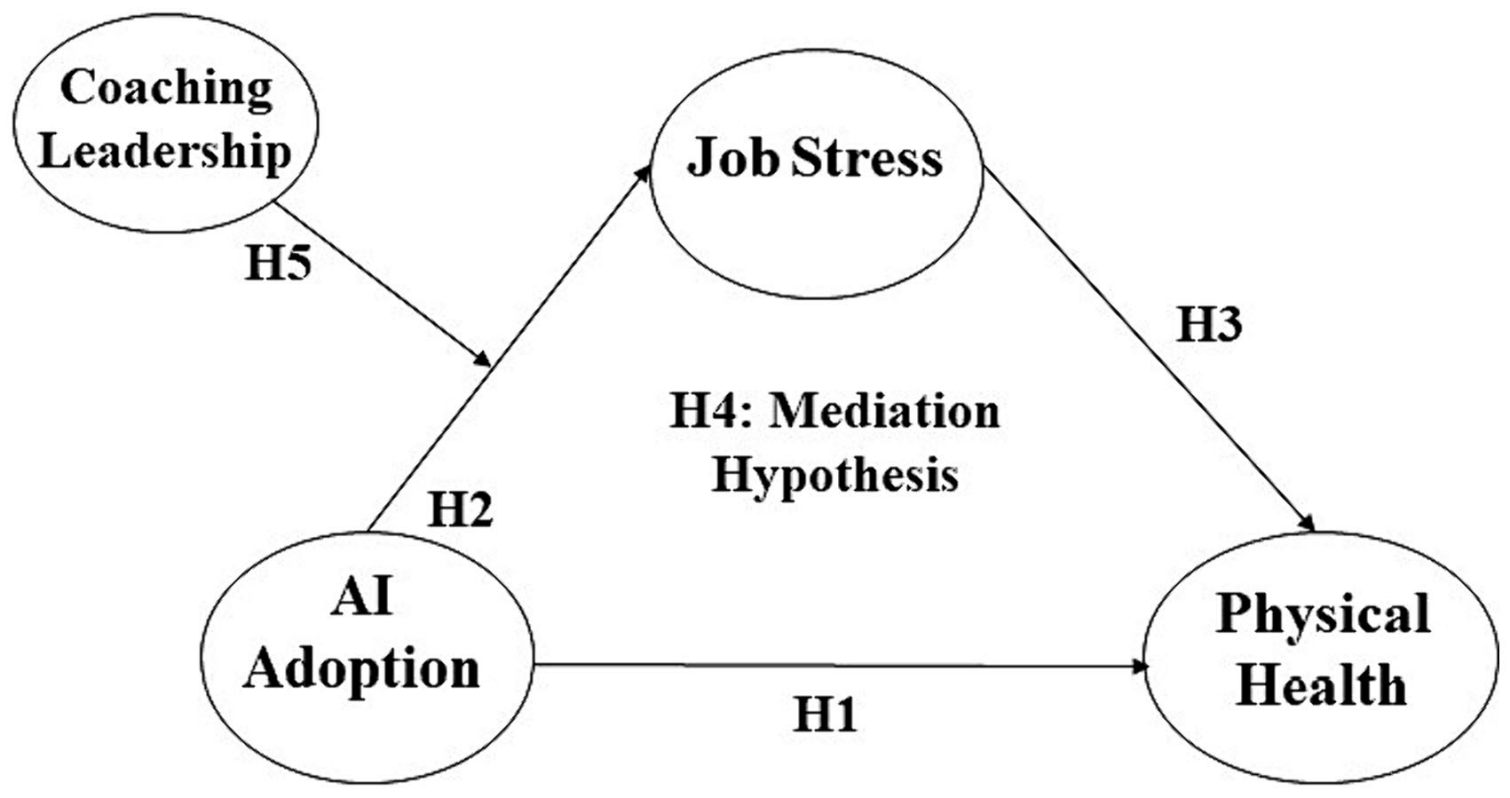
Theoretical model.

The influence of artificial intelligence (AI) adoption on employee physical health can be explained through various theoretical frameworks that shed light on the potential physiological repercussions of technological integration in the workplace. The academic discourse on this subject is underpinned by several theories that collectively provide a robust conceptual scaffold for understanding these dynamics (Figure 1).

First, the biopsychosocial model posits that physical health is a result of a complex interplay between biological, psychological, and social factors. AI adoption can influence these factors by altering work environments and job demands, which in turn can affect stress levels and health behaviors, leading to physiological changes (37). This perspective provides a broader framework considering how occupational stressors associated with AI, like job redesign or deskilling, can impact health through chronic stress, leading to long-term health conditions (38).

Second, the technostress theory also has implications for physical health. The stress arising from adapting to new AI technologies can lead to physiological symptoms such as headaches, fatigue, and gastrointestinal disturbances, as indicated by Brod (39) and later by Tarafdar et al. (40). Job strain is a function of work demands and the amount of control an employee has over their work tasks. AI can change the nature of job demands and control, potentially increasing strain and associated health risks like cardiovascular disease (41).

Third, the conservation of resources (COR) Theory of Hobfoll (26) is relevant in this context as well, considering that the loss of resources (e.g., job security, social support) due to AI can trigger stress responses that have a deleterious impact on physical health, such as immunosuppression or hypertension (26). According to the COR theory proposed by Hobfoll (26), stress is fundamentally a response to the environment where there is a threat of resource loss, actual resource loss, or inadequate resource gain following investment. In the context of AI adoption, employees might perceive a threat to their valuable resources, including job competency, autonomy, and a sense of security due to the introduction of advanced, potentially job-altering technologies. These perceived threats or actual losses can catalyze stress responses, which, if persistent, may lead to physical health detriments, as the theory posits that prolonged stress can have tangible physiological effects. For instance, employees may fear skill obsolescence as AI might take over tasks they were previously responsible for, or they might experience a reduction in social support as AI-driven processes become more prevalent, potentially leading to isolation at work. In this situation, the employees might need to invest additional resources, such as time and effort, to adapt to or learn new AI systems, which can be particularly taxing if these investments do not lead to adequate returns in terms of enhanced job security, performance, or satisfaction. This imbalance, as outlined by COR theory, could exacerbate stress and, subsequently, adversely affect physical health.

Each of these theories provides a lens through which the potential impact of AI on employee physical health can be examined, offering a theoretical and empirical foundation for further research and practical intervention.

*Hypothesis 1*: AI adoption in organizations will lead to a decrease in employee physical health.

### The impact of AI adoption on job stress

Introduced by Hobfoll (26), the Conservation of Resources (COR) theory has become an influential framework for understanding stress within the organizational context. The theory postulates that individuals strive to obtain, maintain, and safeguard valued resources, both tangible and intangible. According to Hobfoll (26), stress is not merely a reaction to a particular stimulus but rather a reaction to resource loss, the threat of loss, or the lack of expected resource gain. As such, any factor that threatens the conservation of these resources can be a potential source of stress.

With the increasing adoption of artificial intelligence (AI) in organizational processes, there arises a new dimension of challenges and demands placed upon employees. AI can introduce complexities by altering task structures, demanding new skills, and shifting organizational dynamics (20). These rapid changes may be perceived as threats to employees’ resources, such as their existing skills, job security, or comfort with established routines (17). From the COR theory’s perspective, these threats can lead to potential or actual resource loss (42), thereby contributing to increased job stress.

Artificial Intelligence adoption can also amplify stress through mechanisms like role ambiguity, enhanced performance expectations, or fears of being replaced by technology (43). Employees might be tasked with understanding complex AI-driven tools, leading to cognitive overload and fears about keeping pace with technological advancements (6, 17, 19). Moreover, the very nature of AI, which is designed to optimize and sometimes outperform human capabilities, might intensify perceptions of resource inadequacy among employees (17).

It is also worth noting that while AI adoption can pose challenges, it simultaneously offers a suite of opportunities (6). AI can eliminate mundane tasks, provide insights through data analysis, and optimize operations (15, 30). However, the dual-edged nature of AI, which provides opportunities while introducing new demands, can lead to a juxtaposition of resource gain and resource threat, further complicating the stress dynamics (6). Employees may benefit from AI’s capabilities while concurrently feeling threatened by its demands, thus accentuating the stress process (44).

In addition, the technostress theory and job demands-resources (JD-R) model also support our hypothesis. Technostress refers to the stress individuals experience due to their inability to cope with new technologies. Tarafdar et al. (40) extended this theory to explain how technology can lead to role overload, invasion of privacy, role ambiguity, and job insecurity, which are significant stressors in the context of AI adoption. Employees may feel overwhelmed by continuous learning requirements and the fast pace of technological change (40). And, the job demands-resources (JD-R) model: The JD-R model, developed by Demerouti et al. (45), posits that job demands (physical, social, or organizational aspects of the job that require sustained effort) can lead to exhaustion and stress. AI can introduce new demands, such as increased complexity of tasks and the need for new skills, potentially elevating stress levels (45).

Incorporating the aforementioned theoretical discourse and anchoring it within the COR framework, we propose the following hypothesis:

*Hypothesis 2*: AI adoption in organizations will lead to an increase in employee job stress.

### The influence of employee job stress on physical health

Central to COR is the proposition that individuals are motivated to protect, retain, and build resources, and stress arises when these resources are threatened, lost, or when investment in resources does not lead to anticipated gains. Physical health, being an invaluable resource, becomes susceptible to the pernicious effects of prolonged job stress, as suggested by the tenets of this theory. Job stress manifests when employees perceive an imbalance between their job demands and the resources, they possess to address those demands (42). Such stressors can be consistent or episodic, but over time, they lead to a gradual erosion of personal resources, including energy, resilience, and even physical health. The depletion of these resources does not merely stop at the cognitive or emotional levels. The constant strain and lack of recuperation begin to manifest in physical ailments, resulting from the body’s continual operation in a heightened state of alertness (35, 36, 46).

When individuals experience persistent job stress, there are identifiable physiological responses. Chronic stress activates the body’s stress response system, leading to prolonged exposure to cortisol and other stress hormones (47). This hormonal imbalance can result in a cascade of adverse health outcomes, including compromised immune function, cardiovascular diseases, metabolic syndrome, and other stress-induced disorders. Moreover, prolonged stress might also lead to maladaptive behaviors such as poor sleep patterns, unhealthy eating habits, and reduced physical activity, further exacerbating the decline in physical health. It is paramount to recognize that the deleterious impact of job stress on physical health is typically cumulative (48, 49). The COR theory suggests that the loss of resources in one domain can lead to a spiral of resource loss in other domains, termed as “loss spirals” (29). In the context of job stress and physical health, sustained periods of stress not only deplete psychological resources but also, over time, erode physical vitality, leading to pronounced physical health declines. Drawing from the above rationales, we posit the following hypothesis:

*Hypothesis 3*: Job stress will decrease employee physical health.

### The mediating effect of job stress in the AI adoption-physical health link

While the Conservation of Resources (COR) Theory (26) fundamentally sheds light on the motivation to preserve, protect, and accumulate resources, it also alludes to the intricate pathways through which resource loss or threats to resources lead to discernible outcomes. Resources are not just tangible commodities; they encompass a broad spectrum that includes personal characteristics, conditions, energies, and objects (50). The mediating effects, in COR’s purview, capture the sequential processes that delineate how resource threats or losses in one domain (e.g., adoption of AI at work) manifest in outcomes in another domain (e.g., physical health).

Adopting AI within organizational settings, as articulated previously, can be perceived as a challenge or threat to an individual’s existing resources, especially in terms of skills or established work routines (51, 52). This perceived threat instigates a stress response, given that employees might feel they are ill-equipped to handle the technological shifts, or that the AI systems might render their skills obsolete. In the lens of COR, AI adoption can pose a threat to an individual’s sense of job security, competency, and predictability, thereby siphoning off their cognitive and emotional resources (17, 50).

Job stress, in the context of COR, can be construed as an attitudinal response signifying the perceived or actual loss of resources (42). When faced with AI-driven changes, employees might grapple with role ambiguity, cognitive overload, and feelings of inadequacy (1). This heightened stress, an aggregate of the negative attitudinal responses, does not remain confined to the cognitive or emotional domain. As proposed by Sauter and Hurrell (53), prolonged job stress can take a toll on one’s physical health; given that the body’s persistent state of arousal in response to stressors can lead to wear and tear over time. The mediating role of job stress elucidates a pathway through which AI adoption translates into adverse physical health outcomes. Drawing from the COR framework, the adoption of AI can be seen as initiating a loss spiral (50), where the initial threat to resources (due to AI adoption) manifests as increased job stress, which further exacerbates resource depletion, culminating in physical health deterioration (27). Anchored in the foundational tenets of COR theory and the interplay between AI adoption, job stress, and physical health, we formulate the following hypothesis:

*Hypothesis 4*: Employee job stress will mediate the relationship between AI adoption and employee physical health.

### The moderating role of coaching leadership in the AI adoption-job stress link

The Conservation of Resources (COR) Theory, as expounded by Hobfoll (26), underscores the fundamental human motivation to protect and accumulate resources. While resources can be tangible, the theory also identifies intangibles such as social support, self-esteem, and knowledge as critical assets that individuals strive to retain. These resources play an instrumental role in buffering against potential threats or actual losses (27). As established, the advent of AI in organizational landscapes presents employees with novel challenges, potentially perceived as threats to their established skills and competencies (44). These changes might propel individuals into situations where they feel ill-equipped, thereby posing a threat to their resources, and resulting in heightened job stress.

In the COR perspective, leadership, particularly coaching leadership, is a potent organizational resource. Coaching leadership emphasizes guidance, mentoring, and fostering a supportive environment (25, 31, 32). Such leadership empowers employees with the necessary knowledge, boosts their self-efficacy, and offers them the emotional and strategic support needed to navigate the challenges introduced by AI (24, 32, 54). Through these mechanisms, coaching leadership acts as a reservoir of resources for employees (54), replenishing what might be lost due to AI-induced demands.

Given the protective and restorative nature of coaching leadership, it stands to reason that its presence can attenuate the potential stress induced by AI adoption. In environments where coaching leadership is pronounced, the resources offered by such leadership can cushion the blow of AI-related changes, mitigating the resultant stress (31, 32, 54). Conversely, in settings where coaching leadership is absent or lacking, the strain of AI adoption may be felt more acutely by employees (54). Drawing from the insights provided by the COR theory and recognizing the pivotal role of coaching leadership as a resource, we propose the following hypothesis:

*Hypothesis 5*: Coaching leadership will moderate the positive relationship between AI adoption and employee job stress, such that the relationship will be attenuated when coaching leadership is high compared to when it is low.

## Methods

### Participants and procedure

The research cohort encompassed working professionals aged above 20 from various South Korean corporations. Engagements with these individuals were orchestrated at three distinct temporal junctures. Recruitment was facilitated through a leading online research entity, boasting an extensive participant pool of roughly 4,390,000 registrants. During registration, these respondents delineated their occupational affiliations online. This research firm also implemented a user verification protocol, necessitating participants to provide contact details like mobile numbers or electronic mail addresses. Leveraging online survey methodologies is recognized as an efficacious strategy for securing a heterogeneous participant sample (55).

Data procurement was pursued from actively employed South Korean professionals across three different timelines, an approach envisioned to circumvent the inherent limitations of cross-sectional data collection. The digital infrastructure of this system empowered the research team to meticulously monitor and ascertain consistent participation of respondents over these timelines. Survey engagements were scheduled at intervals spanning 5–6 weeks and remained accessible for a span of 2–3 days, granting ample opportunity for participants to render their feedback. Integral to the data collection process, the research entity implemented rigorous mechanisms to deter geo-IP discrepancies and identify anomalously swift responses.

Outreach efforts to solicit survey participation were directly managed by the research entity. Prospective participants were provided assurances of the voluntary nature of their involvement, with a commitment to maintaining the confidentiality of their submissions, restricted solely to research pursuits. Those who opted to participate were duly enlightened regarding the study’s parameters and their explicit consent was acquired, ensuring full adherence to ethical standards. A monetary incentive, ranging between $9 and 10, was proposed as a token of gratitude for their contribution.

To attenuate potential sampling distortions, the research entity deployed a stratified random sampling technique. This strategy entailed random participant extraction from each predefined category, thereby attenuating biases potentially arising from demographics, professional standing, academic credentials, or industry affiliation. Through intricate online tracking mechanisms, the entity ensured consistent participation of the same respondents across all three temporal data collection phases.

In the inaugural data collection phase, 718 professionals responded; the subsequent phase saw 507 responses; and the final juncture witnessed 378 responses. Post-collection, the dataset underwent a purification process, wherein incomplete responses were excised. The culminating dataset, deemed fit for research analysis, comprised 375 respondents who provided comprehensive responses throughout the three survey stages, yielding a response efficacy of 52.23%. The determinant for this sample size was influenced by past scholarly recommendations, involving considerations like the G*Power statistical evaluation for optimal sample size (56).

Conclusively, the three-wave time-lagged research paradigm was embraced to surmount potential shortcomings observed in extant literature, proffering a more resilient and precise discernment of causal interrelations amidst variables. This model’s unique strength lies in its capacity to capture data at three differentiated timelines, facilitating an in-depth analysis of temporal sequencing while concurrently addressing confounding elements. It further paves the way for an understanding of potential reciprocal dynamics between variables under consideration. Through establishing unambiguous temporal sequences, this paradigm redresses issues typically observed with cross-sectional designs, which occasionally grapple with ascertaining causality and might be susceptible to common methodological biases. The tripartite design also provides a refined grasp of causal underpinnings delineating variable interplay, thereby augmenting both the internal validity and applicability of conclusions. This, in turn, fortifies the overarching academic discourse with increased rigor and credibility (Table 1).

**Table 1:** Descriptive characteristics of the sample.

Characteristic	Percent
Gender	
Men	51.2%
Women	48.8%
Age (years)	
20–29	21.9%
30–39	22.7%
40–49	28.0%
50–59	27.5%
Education	
High school or below	12.8%
Community college	18.9%
Bachelor’s degree	56.0%
Master’s degree or higher	12.3%
Position	
Staff	42.1%
Assistant manager	16.3%
Manager or deputy general manager	24.0%
Department/general manager or director and above	17.6%
Industry type	
Manufacturing	22.9%
Services	14.9%
Construction	11.2%
Health and welfare	14.9%
Information services and telecommunications	9.8%
Education	16.5%
Financial/insurance	2.7%
Consulting and advertising	0.8%
Others	7.2%
Firm size	
1–9 employees	15.5%
10–29 employees	18.4%
30–49 employees	10.9%
50–99 employees	14.1%
100–149 employees	7.7%
150–299 employees	6.7%
300–449 employees	3.5%
500–999 employees	7.2%
1,000–4,999 employees	9.6%
Above 5,000 employees	6.4%

### Measures

At time point 1, the participants were asked about their levels of AI adoption and coaching leadership. And, time point 2, they were asked to evaluate their degrees of job stress. At the last point, data about the degree of their physical health were collected. All the variables were measured via multi-item scales on a five-point Likert scale.

### AI adoption (time point 1, collected from employees)

To measure the extent of AI adoption in an organization, we utilized five items from established studies by adapting the scales (50, 57, 58). The items in our study included the following: “Our company uses artificial intelligence technology in its human resources management system,” “Our company uses artificial intelligence technology in its production and operations management systems,” “Our company uses artificial intelligence technology in its marketing and customer management systems,” “Artificial intelligence technology is used in our company’s strategic and planning systems,” and “Artificial intelligence technology is used in our company’s (organization’s) financial and accounting systems.” The Cronbach’s alpha value was 0.92.

### Coaching leadership (time point 1, collected from employees)

To measure the degree of coaching leadership, we utilized 12 items from previous studies on coaching leadership (31, 32). Sample items were: “My leader believes in my potential for growth,” “When a situation needs my leader’s experiences, he/she willingly discusses them,” “In discussion with me, my leader focuses on my individual needs,” “My leader views differences of opinion as constructive,” “To improve work performance, my manager constantly provides feedback,” “In order to improve my performance, my manager serves as a role model,” and “My leader asks questions that make me reflect on my thoughts and perspectives.” The Cronbach’s alpha value was 0.94.

### Job stress (time point 2, collected from employees)

To evaluate the degree of job stress, we utilized four items of the job stress scale by adapting the scales of previous studies (36, 46). The items in our study included the following: “I feel a great deal of stress because of my job,” “I experience a fair amount of stressful events at work,” “My job is quite stressful,” “I feel frustrated at work,” “I feel tense or anxious at,” and “I feel angry or irritated at work.” The value of Cronbach’s alpha in this research was 0.90.

### Physical health (time point 3, collected from employees)

This study used eight items from the physical health scale, which were utilized in the previous works (48, 49, 59). The items in our study included the following: “How would you describe your recent overall physical health?,” “Compared to people your age, how is your physical health?,” “In your opinion, how is your physical health?,” “How are your health conditions related to high blood pressure?,” “How are your health conditions related to diabetes or blood sugar?,” “How about health conditions related to heart attack, angina, myocardial infarction, and congestive heart failure?,” “What about health conditions related to cerebrovascular diseases (e.g., stroke, cerebral hemorrhage, and cerebral infarction)?,” and “How are your health conditions related to arthritis or rheumatism?.” The Cronbach’s alpha value was 0.87.

### Control variables

Taking into account the recommendations of prior research (48, 49), this study accounted for the effects of physical health by including various control variables, including age, industry/sector, gender, education, and position. These variables were collected at time point 1.

### Statistical analysis

A correlational examination was executed utilizing SPSS 26.0 program to ascertain the interrelationships among the selected variables. In line with the methodological recommendations proposed by Anderson and Gerbing (60), the research adopted a bifurcated procedure comprising both a measurement and a structural model. The validity of the measurement model was established through a Confirmatory Factor Analysis (CFA). Subsequently, the structural model’s assessment entailed a moderated mediation model examination, undertaken using the AMOS 26.0 software, deploying the maximum likelihood (ML) estimator, consistent with Structural Equation Modeling (SEM) tenets.

To ascertain the congruence of the formulated model, various indices assessing the goodness-of-fit were employed. These encompassed the comparative fit index (CFI), the Tucker–Lewis index (TLI), and the root mean square error of approximation (RMSEA). Drawing from extant literature, it is posited that ideal threshold values for both CFI and TLI should exceed 0.90, while for RMSEA; it ought to remain below 0.06. In a subsequent phase, a bootstrapping technique was employed to gage the relevance of the intermediary effect, as delineated by Shrout and Bolger (61). The research incorporated bootstrapping analysis set at a 95% bias-adjusted confidence interval (CI) to validate the mediation proposition. Should the CI exclude the value 0, it is indicative of the intermediary effect achieving statistical significance at the 0.05 level, corroborating the assertions of Shrout and Bolger (61).

## Results

### Descriptive statistics

Our research found that the variables of AI adoption, job stress, and physical health were strongly correlated. The results of the correlation analysis are displayed in Table 2.

**Table 2:** Correlation among research variables.

	Mean	S.D.	1	2	3	4	5	6	7
1. Gender_T1	1.49	0.50	-						
2. Education_T1	2.68	0.85	−0.07	-					
3. Tenure_T1	67.43	73.39	−0.10	0.04	-				
4. Position_T1	2.42	1.51	−0.38^**^	0.28^**^	0.32^**^	-			
5. AIA_T1	2.27	0.99	−0.18^**^	0.12^*^	0.03	0.07	-		
6. CL_T1	3.27	0.61	−0.14^**^	0.15^**^	0.01	0.13^*^	0.19^**^	-	
7. JS_T2	2.84	0.85	−0.01	0.13^*^	−0.08	0.01	0.19^**^	−0.13^*^	-
8. PH_T3	3.62	0.32	0.06	0.04	0.01	0.04	0.02	0.13^*^	−0.23^**^

^*^p < 0.05. ^**^p < 0.01. S.D. means standard deviation, AIA means AI adoption, CL means coaching leadership, JS means job stress, and PH indicates physical health. As for gender, males are coded as 1 and females as 2. As for position, general manager or higher are coded as 5, deputy general manager and department manager as 4, assistant manager as 3, clerk as 2, and others below clerk as 1. As for education, “below high school diploma” level is coded as 1, “community college” level as 2, “bachelors” level as 3, and “master’s degree or more” level is coded as 5.

### Measurement model

We tested the discriminant validity of the four main research variables (AI adoption, coaching leadership, job stress, and physical health) by performing a confirmatory factor analysis (CFA) of all items to evaluate the goodness-of-fit of the measurement model. The result of the CFA showed that the model fit indices of the four-four model were good enough [(df = 141) = 241.528, CFI = 0.979, TLI = 0.974, and RMSEA = 0.044]. Then, a series of chi-square difference tests were implemented through comparing the four-factor model (AI adoption, coaching leadership, job stress, and physical health) to other alternative models including the three-factor [χ^2^ (df = 144) = 721.343, CFI = 0.877, TLI = 0.854, and RMSEA = 0.104], two-factor [χ^2^ (df = 146) = 1493.718, CFI = 0.712, TLI = 0.663, and RMSEA = 0.157], and one-factor [χ^2^ (df = 147) = 2207.605, CFI = 0.560, TLI = 0.488, and RMSEA = 0.194] model. The series of chi-square different tests illustrated that the four-factor model outperformed its counterparts in terms of fit. Most of all, the results solidified that the four research variables upheld appropriate discriminant validity.

### Structural model

Within the ambit of this manuscript, a moderated mediation paradigm was devised to probe our hypotheses. This theoretical paradigm amalgamated both mediation and moderation structures. From the mediation standpoint, the influence of AI adoption on physical health was mediated through the level of job stress. In addition, in the moderation framework, coaching leadership functioned as a contextual variable, mitigating the increasing influence of AI adoption on job stress.

In this moderation framework, the interaction term amalgamating AI adoption and coaching leadership was forged by their multiplication. To temper the repercussions of multi-collinearity, an initial centering of variables around their means was executed. Such a centering method not merely curtailed the prevalence of multi-collinearity but also safeguarded against correlation diminution, thereby fortifying the moderation analysis’s veracity (62).

To quantify potential multi-collinearity distortion, the present inquiry gaged variance inflation factors (VIF) and associated tolerances, as expounded by Brace et al. (62). Both AI adoption and coaching leadership manifested VIF metrics at 1.038. Similarly, tolerance indices stood at 0.963 for both aforementioned variables. This analytic output insinuated that both AI adoption and coaching leadership remained largely insusceptible to multi-collinearity challenges, given the VIF metrics remained beneath 10 and tolerance metrics surpassed 0.2.

### Results of the mediation analysis

To ascertain the optimal mediation model, a chi-square different test was undertaken to juxtapose a full mediation structure against its partial counterpart. The full mediation design was congruent to its partial version, barring the overt linkage between AI adoption and physical health. Both the full and partial mediation structures exhibited satisfactory fit, as manifested by the fit indices [χ^2^ = 358.459 (df = 189), CFI = 0.961, TLI = 0.951, and RMSEA = 0.049] for the full mediation model, and [χ^2^ = 356.131 (df = 188), CFI = 0.961, TLI = 0.952, and RMSEA = 0.049] for the partial one.

Yet, the output of the chi-square different analysis propounded that the full mediation structure was more befitting, as evinced by the chi-square difference [Δχ^2^ (1) = 2.328, *p* > 0.05, non-significant] among the mediation models. This indicates that AI adoption exerted an indirect influence, rather than a direct one, on physical health via the intermediary effect of job stress. The study further incorporated control variables within the model, encompassing elements like tenure, gender, educational background, and occupational position concerning physical health. The analytic outcome showed that all control variables (age, industry/sector, gender, education, and position) could not reach statistical significance.

Our investigative framework, which amalgamated the control variables, divulged that AI adoption has a non-significant association with physical health (β = −0.01, *p* > 0.05), leading to the negation of Hypothesis 1. The partial mediation model, delineating a trajectory from AI adoption to physical health, manifested an insignificant coefficient, further underscoring the superiority of the full mediation model, which was accepted as the terminal one. Such an observation corroborates the preeminence of the full mediation model over its partial counterpart, bolstered by enhanced fit indices. Given the juxtaposition of the full and partial mediation models, coupled with the non-significant path from AI adoption to physical health, Hypothesis 1 is hereby dismissed. The conclusion underscores that the ramifications of AI adoption for physical health are conceivably mediated through elements such as job stress rather than a direct pathway.

**Figure 2:**
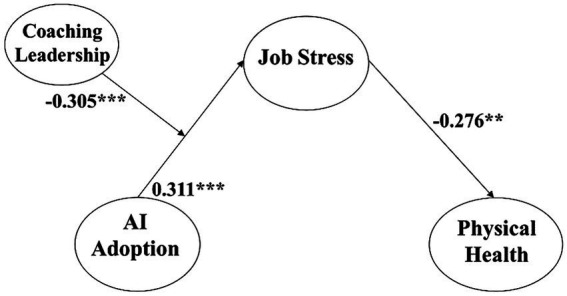
Coefficient values of our research model (^***^p < 0.001. All values are standardized).

The analytic outcomes corroborate both Hypotheses 2, elucidating that AI adoption exerts a substantial and escalating impact on job stress (β = 0.311, *p* < 0.001) and Hypothesis 3 that job stress decreases physical health in a significant way (β = −0.276, *p* < 0.01). These deductions are visually represented in Table 3 and Figure 2.

**Table 3:** Results of structural model.

Hypothesis	Path (Relationship)	Unstandardized estimate	S.E.	Standardized estimate	Supported
1	AI adoption → Physical health	−0.012	0.009	−0.085	No
2	AI adoption → Job stress	0.230	0.039	0.311^***^	Yes
3	Job stress → Physical health	−0.052	0.018	−0.276^**^	Yes
5	AI adoption × Coaching Leadership	−0.325	0.054	−0.305^***^	Yes

^**^p < 0.01. ^***^p < 0.05. Estimate indicates standardized coefficients. S.E. means standard error. The coefficient value of the path from AI adoption to physical health (H1) was in the partial mediation model, which was not accepted as a final model.

### Bootstrapping

In an endeavor to validate Hypothesis 4—which postulates that job stress could potentially act as a mediator between AI adoption and physical health—the research adopted a bootstrapping approach, utilizing a substantial sample of 10,000. This procedure aligned with the methodology delineated by Shrout and Bolger (61). For the indirect effect emanating from job stress to be deemed statistically significant, it is imperative that the 95% bias-corrected confidence interval (CI) pertaining to the average indirect effect eschew the inclusion of the value zero, as per the guidance of Shrout and Bolger (61).

Further, to meticulously assess the mediating role of job stress in linking AI adoption to physical health (as posed in Hypothesis 4), the investigation employed a bootstrapping technique with the aforementioned sample size. The statistical significance of this indirect mediation effect is affirmed when the 95% bias-corrected CI pertaining to this average effect precludes the value of zero. The findings from this analysis yielded a CI that distinctly did not encompass zero (95% CI = [−0.136, −0.048]), conclusively signifying that the mediating role of job stress holds statistical weight. This not only solidifies the claims made in Hypothesis 4 but also further accentuates the intricate connections. The comprehensive delineation of the direct, indirect, and total effects of the trajectories originating from AI adoption on physical health is meticulously presented in Table 4.

**Table 4:** Direct, indirect, and total effects of the final research model.

Model (Hypothesis 4)	Direct effect	Indirect effect	Total effect
AI adoption → Job stress → Physical health	0.000	−0.086	−0.086

All values are standardized.

### Result of moderation analysis

**Figure 3:**
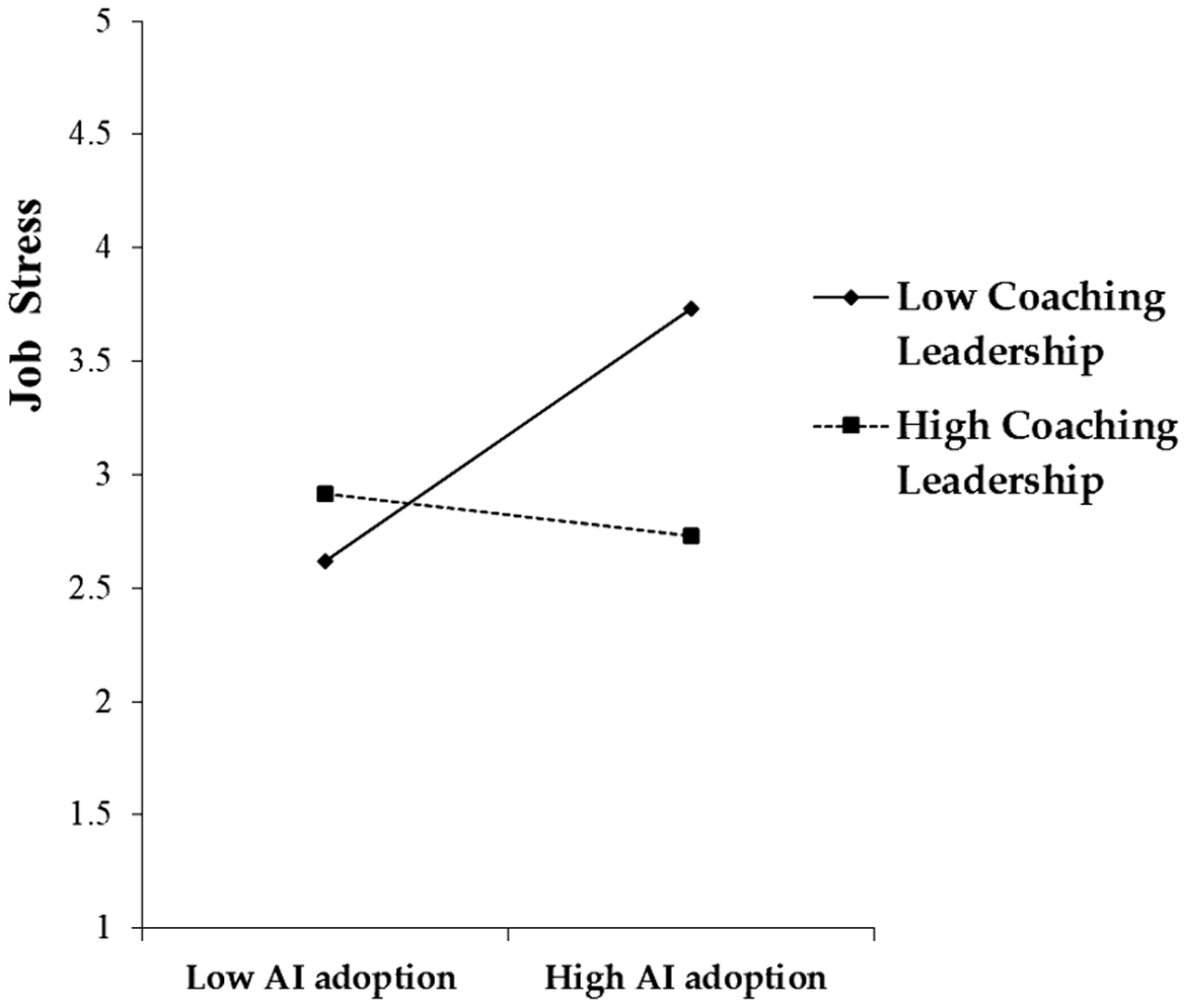
Moderating effect of coaching leadership in the AI adoption–job stress link.

Central to this research was the objective discerning the potential moderating influence of coaching leadership on the connection between AI adoption and job stress. This was methodically realized by fabricating an interaction component that merges both AI adoption and coaching leadership, facilitated through a process of mean-centering. Upon rigorous analysis, it was evident that this interaction term was significant (β = −0.305, *p* < 0.001). This observation implies that coaching leadership has a negative moderating capacity, mitigating the increasing effect that AI adoption potentially has on job stress. This empirical evidence thus provides substantial credence to Hypothesis 5 (Figure 3).

## Discussion

### Theoretical implications

There are some theoretical implications in our study. First, this research accentuates the multifaceted role of resources within the COR framework, especially in the context of rapidly changing technological landscapes (26). By positioning AI adoption as both a potential threat and an avenue for resource expansion, the study underscores the dynamism and adaptability of the COR theory. This expands the theory’s purview, suggesting that the introduction of novel technologies in organizational settings can be both resource-depleting and resource-providing, contingent on various mediating and moderating factors (27).

Second, our study pushes the boundaries of existing literature on job stress, particularly in the wake of AI adoptions. By postulating and testing the mediating role of job stress between AI adoption and physical health, this research offers a nuanced understanding of stress dynamics in contemporary workplaces. This highlights that job stress is not just a product of traditional job demands but can also arise from disruptions introduced by technological innovations (44).

Third, this study accentuates the paramount importance of leadership, especially coaching leadership, amid technological transformations. While prior research has predominantly focused on individual attributes such as locus of control or core self-evaluation to mitigate the adverse impacts of AI on employees (17, 63), our study emphasizes the significance of leadership, an aspect often overlooked in previous research. Leadership, particularly coaching leadership, emerges as essential not only for organizational directives but also for supporting and guiding employees during these technological shifts (24). By positioning coaching leadership as an instrumental tool that can alleviate the stressors introduced by AI adoption, this study offers fresh insights into the dynamically evolving role of leadership in the current organization. Such a shift encourages scholars to revisit and reassess leadership paradigms, especially in scenarios punctuated by rapid technological disruptions (64).

Fourth, the direct and indirect effects of AI adoption on employees’ physical health, as elucidated in this study, emphasize the need for a broader consideration of outcome variables in organizational research. Previous studies have heavily focused on work-related attitudes and behaviors such as job satisfaction, affective commitment, work engagement, job insecurity, job performance, creativity, and job crafting (6, 9, 18, 19, 50, 65). However, this research underscores the intertwined nature of psychological factors (like job stress) and tangible health outcomes, suggesting a more holistic approach to understanding employee well-being (2, 28).

### Practical implications

Based on our findings, there are some practical implications. First, organizations contemplating the adoption of Artificial Intelligence (AI) should be judicious in their approach. AI is not merely a technological asset; its adoption can have profound implications on employees’ well-being (20). Leaders and decision-makers should be proactive in providing adequate training, resources, and support during AI transitions. Such foresight can ensure that the positive opportunities afforded by AI are harnessed without inadvertently escalating job stress and adversely impacting employee health.

Second, in the ever-evolving technological landscape, leadership styles need to adapt. This research accentuates the salience of coaching leadership in mitigating potential stressors introduced by technological advancements (25). Organizations should invest in leadership development programs that emphasize mentorship, guidance, and support (65). By doing so, businesses can foster a work environment wherein employees feel equipped and empowered to navigate AI-induced challenges.

Third, beyond traditional wellness programs, organizations need to recognize the intertwined nature of psychological and physical health (28). Employee well-being initiatives should be holistic, addressing not just physical health but also potential psychological stressors. Given the demonstrated relationship between AI adoption, job stress, and physical health, comprehensive programs that offer stress management and resilience-building resources can be pivotal (1).

Fourth, with the rapid pace of technological changes, organizations must institute robust feedback mechanisms (66). These can help in continuously evaluating the impact of such changes on employee stress and well-being. Regular employee surveys, focus groups, and open channels of communication can offer insights into potential areas of concern, thereby allowing organizations to recalibrate their strategies in real-time and ensure the well-being of their workforce (17).

### Limitations and future research

First, despite utilizing a time-lagged design in our study, which is generally believed to mitigate some concerns associated with common method variance, the sole reliance on self-report measures might still introduce some degree of CMV (67). While time-lagged designs do attenuate potential biases, they do not entirely eliminate the possibility of CMV. Future studies might consider utilizing multi-source data, incorporating peer evaluations, supervisor assessments, or objective measures where feasible, to further minimize the risk of CMV.

Second, our research primarily explored coaching leadership as a moderating variable, underscoring its significance in attenuating the stressors introduced by AI adoption. However, the organizational landscape is multifaceted, and there could be other moderating variables that might similarly play a crucial role. Future research could delve into alternative moderating influences, such as organizational culture, peer support, or training and development initiatives, to understand their potential in buffering against the negative implications of AI on employee well-being.

Third, our study, while in-depth, was grounded in the Korean context, leveraging data from Korean employees. This cultural and regional specificity implies that the findings, while robust within this context, might be influenced by unique Korean cultural values, work ethics, and organizational norms (68). Given this, it becomes paramount for future research to expand the geographical ambit, incorporating cross-cultural studies to discern the extent to which the relationships observed in our study hold true across different cultural landscapes and to understand the nuances introduced by varied cultural values and norms.

Fourth, we could not assure that the respondents in this study interpreted AI in the same way that the authors and other participants interpret AI. In fact, recent work from McElheran et al. (69) using Census Bureau data in the United States shows that only 6% of firms actually use AI. Ensuring that respondents interpret Artificial Intelligence (AI) in the same way as it is conceptualized in this paper is crucial for the validity and reliability of the findings. The discrepancy in AI interpretation, as highlighted by the work of Brynjolfsson et al. using Census Bureau data, where only a small percentage of firms reported actual AI usage, underscores the importance of clear and consistent definitions in research. Reflecting on the finding from McElheran et al. (69), it is important to acknowledge that the perception and adoption of AI can vary greatly across different organizations and sectors. This variation underscores the need for clear communication and understanding of what AI entails in the context of my research. It also suggests that the prevalence of AI adoption reported in studies may be influenced by how respondents interpret and identify AI within their organizational practices.

Fifth, in the examination of the negative effects of Artificial Intelligence (AI) on employee well-being, controlling for individuals who do not utilize AI in their work is a critical methodological consideration. The absence of such a control group in this study raises important questions regarding the generalizability and specificity of the findings to the impact of AI. However, the relevance and contribution of this research can still be justified through several avenues: (1) This study’s primary objective appears to be the investigation of AI’s specific impacts on employees who are users of such technologies. By concentrating on this cohort, the research provides depth and nuance in understanding how AI integration in work processes affects physical and mental health. The findings contribute valuable insights into the direct consequences of AI usage in the workplace, enriching the discourse on technology’s role in shaping work environments and employee well-being. (2) Although the study does not explicitly include a control group of non-AI users, the implications of AI on employee well-being can still be indirectly inferred by comparing the results with existing baseline data on workplace well-being metrics. Such an approach allows for a nuanced interpretation of how AI adoption may deviate from or align with broader occupational health trends. (3) The study’s exploration of AI’s negative effects, without a control group of non-AI users, still offers theoretical contributions by applying and potentially extending current frameworks on job stress, technological stressors, and their health implications. It may identify unique stressors associated with AI, contributing to the development of theory specifically addressing technological impacts on the workforce. (4) For organizations and practitioners, understanding the specific challenges and stressors linked to AI adoption is crucial for developing targeted interventions. This research provides evidence-based insights into the areas where support, training, and resources are most needed to mitigate the adverse effects of AI on employee health, underscoring its practical relevance. (5) Finally, by documenting the effects of AI on users, this study lays the groundwork for future comparative research that includes non-AI users as a control group. It highlights the necessity for further empirical work to delineate the distinct impacts of AI from other technological and organizational stressors, offering a clear direction for subsequent investigations. In conclusion, while the absence of a control group comprising individuals not using AI in their work is a limitation, the study’s focus on AI users is justified by its contribution to understanding the specific health implications of AI adoption. It offers valuable insights for theory, practice, and future research directions, emphasizing the need for comprehensive strategies to manage the integration of AI in the workplace effectively.

Sixth, this paper investigated the physical effects of Artificial Intelligence (AI) adoption on employees, reliance solely on questionnaire data. While questionnaires are a useful tool for gathering data on a wide range of variables, the assessment of physical health effects necessitates a careful and transparent methodological approach. Clarifying the extent to which objective tests or diagnoses—such as clinical evaluations, physiological measurements (e.g., blood pressure, heart rate variability), or other diagnostic tests in addition to self-reported questionnaire were involved in the study is essential for ensuring the robustness of the research and for accurately conveying the implications of AI adoption on employee well-being. Including such measures can significantly enhance the study’s contribution by providing a more comprehensive understanding of the physical health implications of AI usage. We suggest directions for future research, particularly emphasizing the importance of incorporating objective health assessments to validate and extend the findings.

Lastly, the current paper could not include the information about the occupation and income of participants as control variables. Those would be natural controls since job tasks vary substantially and contribute to differences in well-being and perceptions. The primary empirical concern here is that companies adopting AI are pushing employees harder and/or employ workers in jobs that are more likely to complement AI, so these workers are likely to get the burden of work and already work harder and thus could rate their job lower and stress higher. Occupation or job level fixed effects would help mitigate this possibility, but would still need more work to be taken as causal.

## Conclusion

In conclusion, the present study provides a comprehensive examination of the impact of Artificial Intelligence (AI) adoption on employee physical health within the organizational milieu. It elucidates the indirect effect of AI on health outcomes, mediated by job stress, and the moderating role of coaching leadership in this relationship. Contrary to the direct effect postulated in Hypothesis 1, the findings reveal that AI adoption does not significantly affect employee physical health when considered in isolation. This negation shifts the focus to the underlying psychological mechanisms that mediate technological impacts on health.

Hypotheses 2 and 3 were supported, highlighting a substantial link between AI adoption and increased job stress, and in turn, a negative impact of job stress on physical health. These findings align with the Conservation of Resources (COR) theory, which posits that stress ensues from the threat of resource loss, actual resource loss, or a lack of resource gain following the investment of resources. In the context of AI adoption, it appears that job stress functions as a critical mediator that translates technological changes into health outcomes.

Furthermore, the significant moderating effect of coaching leadership, as supported by Hypothesis 5, underscores the importance of leadership practices in technological transitions. Coaching leadership emerges as a vital resource that can mitigate the stress associated with AI, potentially safeguarding employee health.

The implications of this research are twofold. Practically, it emphasizes the need for organizations to cultivate a supportive leadership style to combat the stressors introduced by AI. Theoretically, it extends the application of COR theory to the domain of AI adoption and employee well-being, providing a framework for understanding the indirect pathways through which technology influences health.

Future research should continue to explore these indirect pathways and examine other potential mediators and moderators. As AI becomes increasingly prevalent in the workplace, understanding the full spectrum of its impact on employees is essential for fostering both organizational effectiveness and employee welfare. It is incumbent upon leaders and policymakers to consider these findings and implement strategies that promote not just technological efficiency but also the holistic well-being of the workforce.

## Data availability statement

The original contributions presented in the study are included in the article/Supplementary material; further inquiries can be directed to the corresponding authors.

## Ethics statement

The studies involving humans were approved by Institutional Review Board of Yonsei University. The studies were conducted in accordance with the local legislation and institutional requirements. The participants provided their written informed consent to participate in this study.

## Author contributions

JJ: Writing – original draft, Software, Methodology, Investigation, Conceptualization. B-JK: Writing – review & editing, Writing – original draft, Visualization, Supervision, Methodology, Formal analysis, Data curation, Conceptualization. JL: Writing – review & editing, Validation, Supervision, Resources, Project administration, Investigation, Funding acquisition, Conceptualization.
