# Supplementary figures and images for: Navigating AI transitions: how coaching leadership buffers against job stress and protects employee physical health
Source: Front Public Health. 2024 Mar 27;12:1343932. doi: 10.3389/fpubh.2024.1343932 (PMC11004349; doi:10.3389/fpubh.2024.1343932)

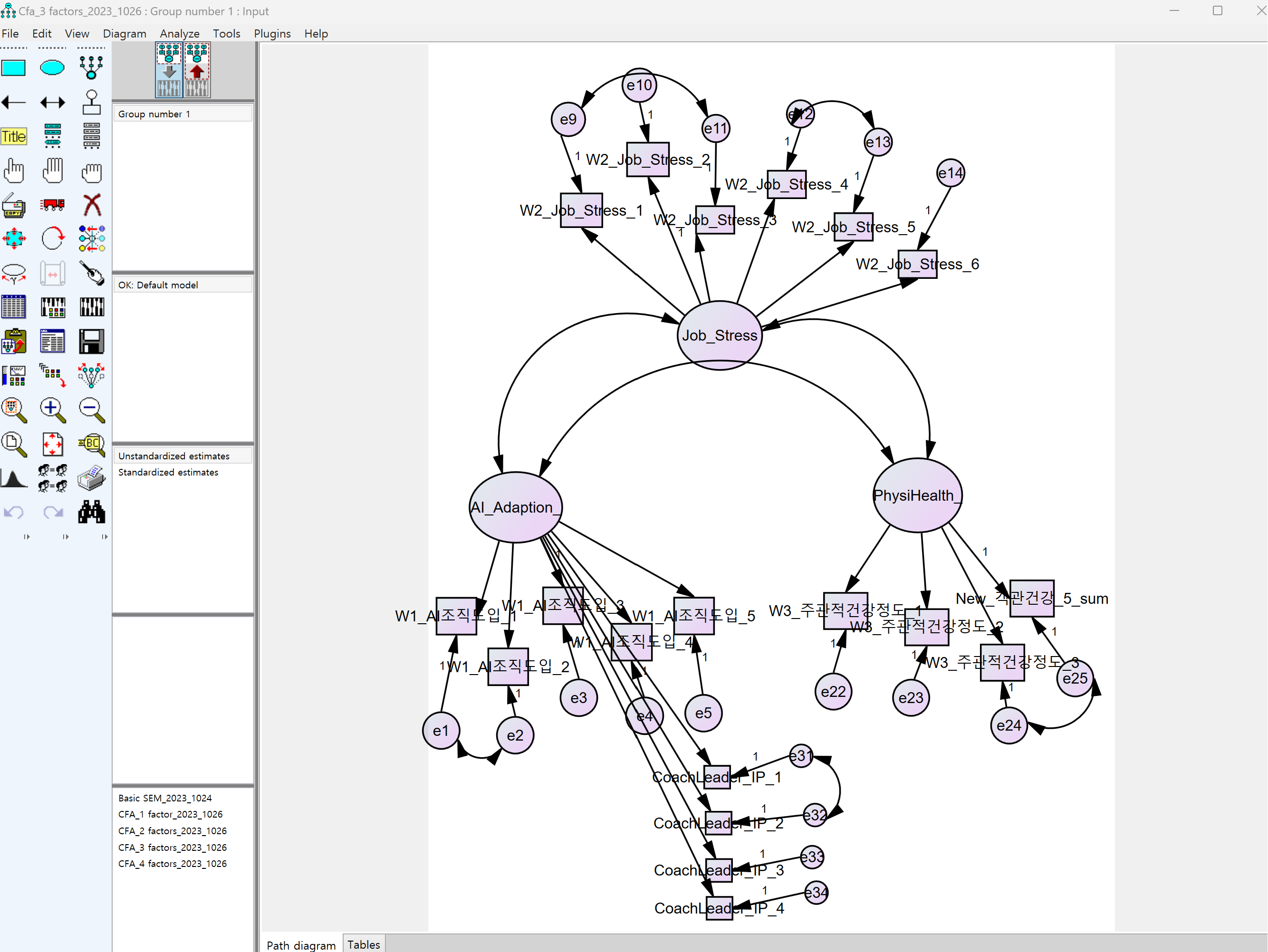

Supplement: Supplementary file 1 [file Image_1.PNG]

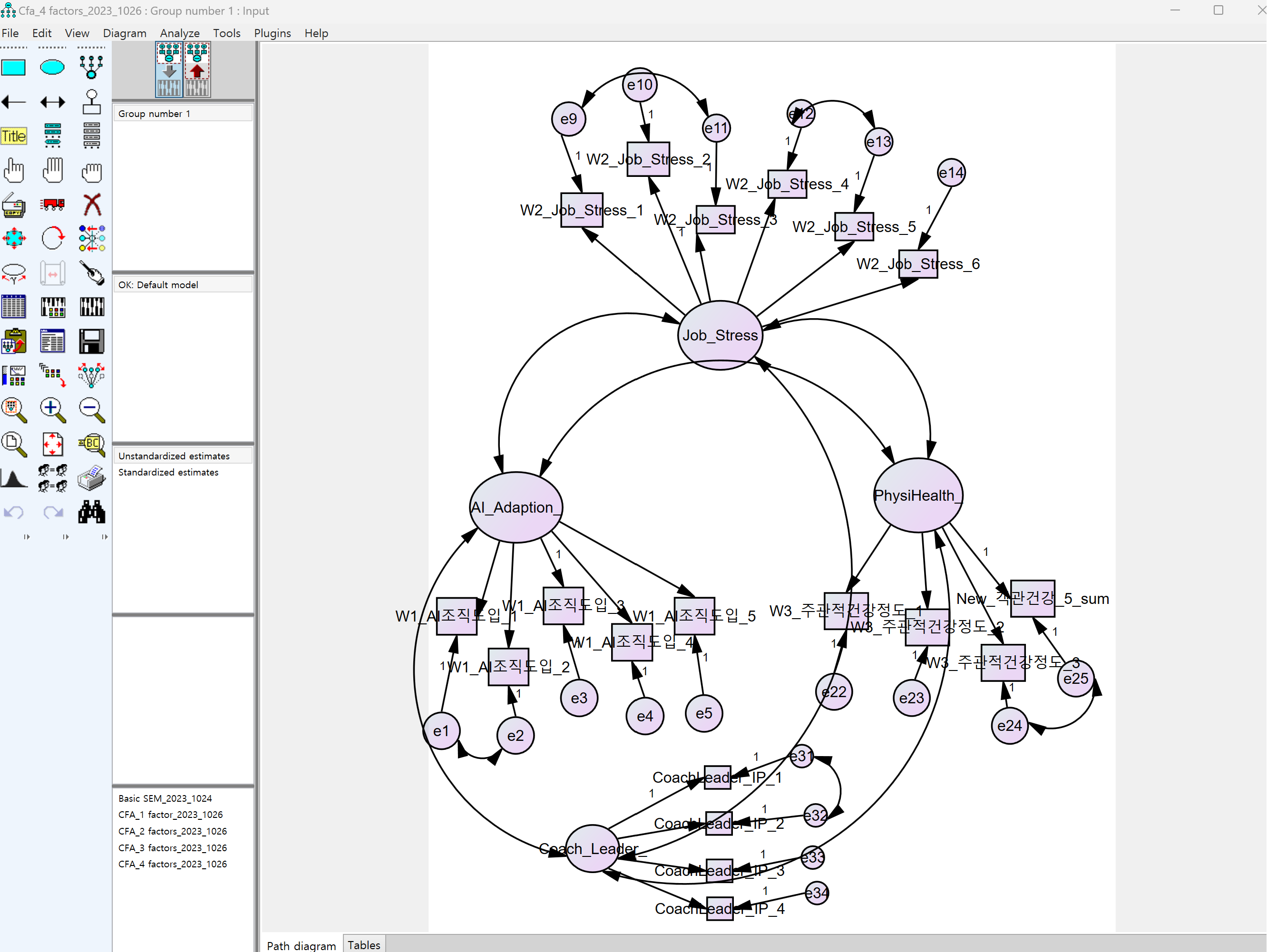

Supplement: Supplementary file 2 [file Image_2.PNG]

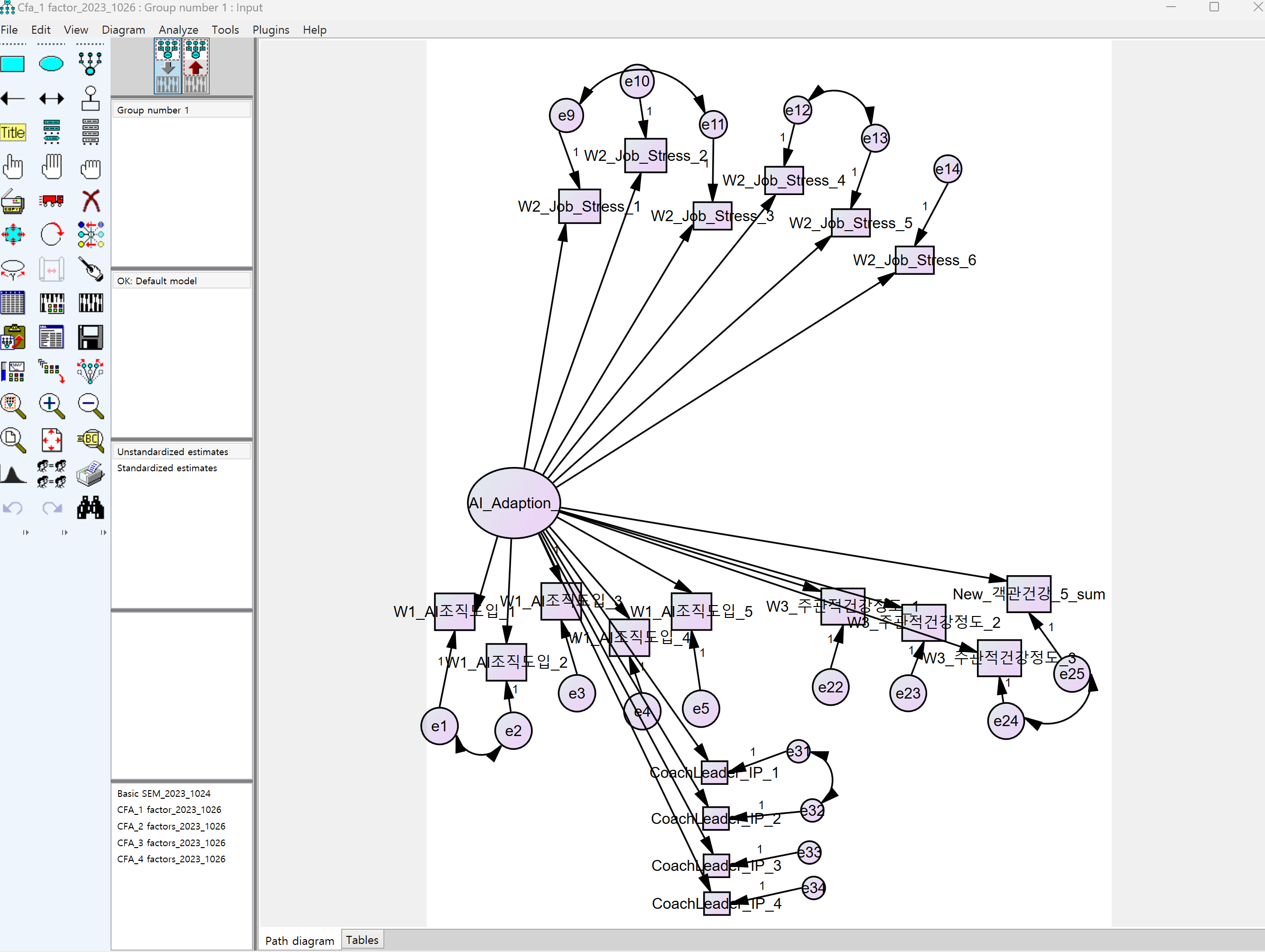

Supplement: Supplementary file 3 [file Image_3.PNG]

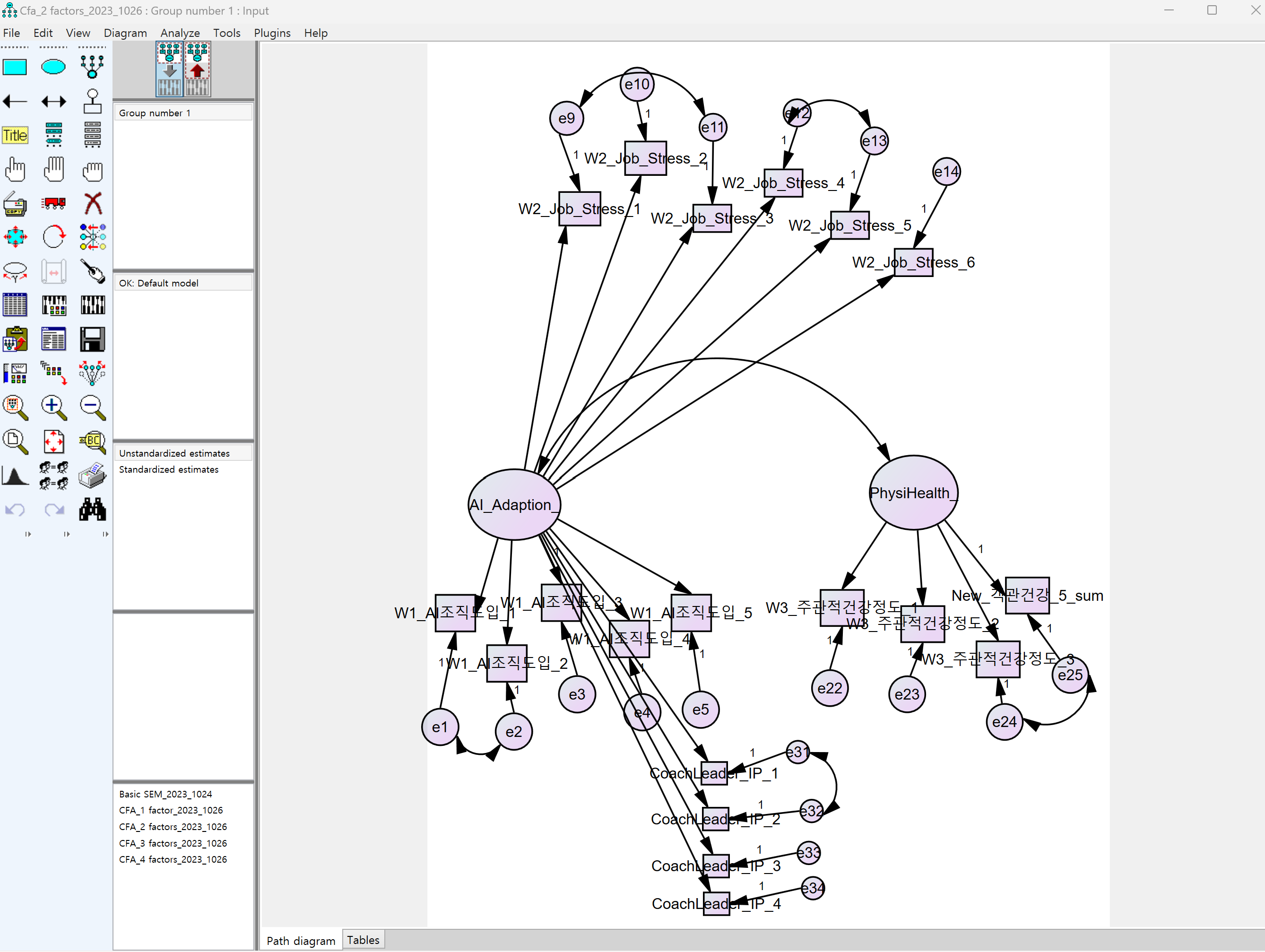

Supplement: Supplementary file 4 [file Image_4.PNG]

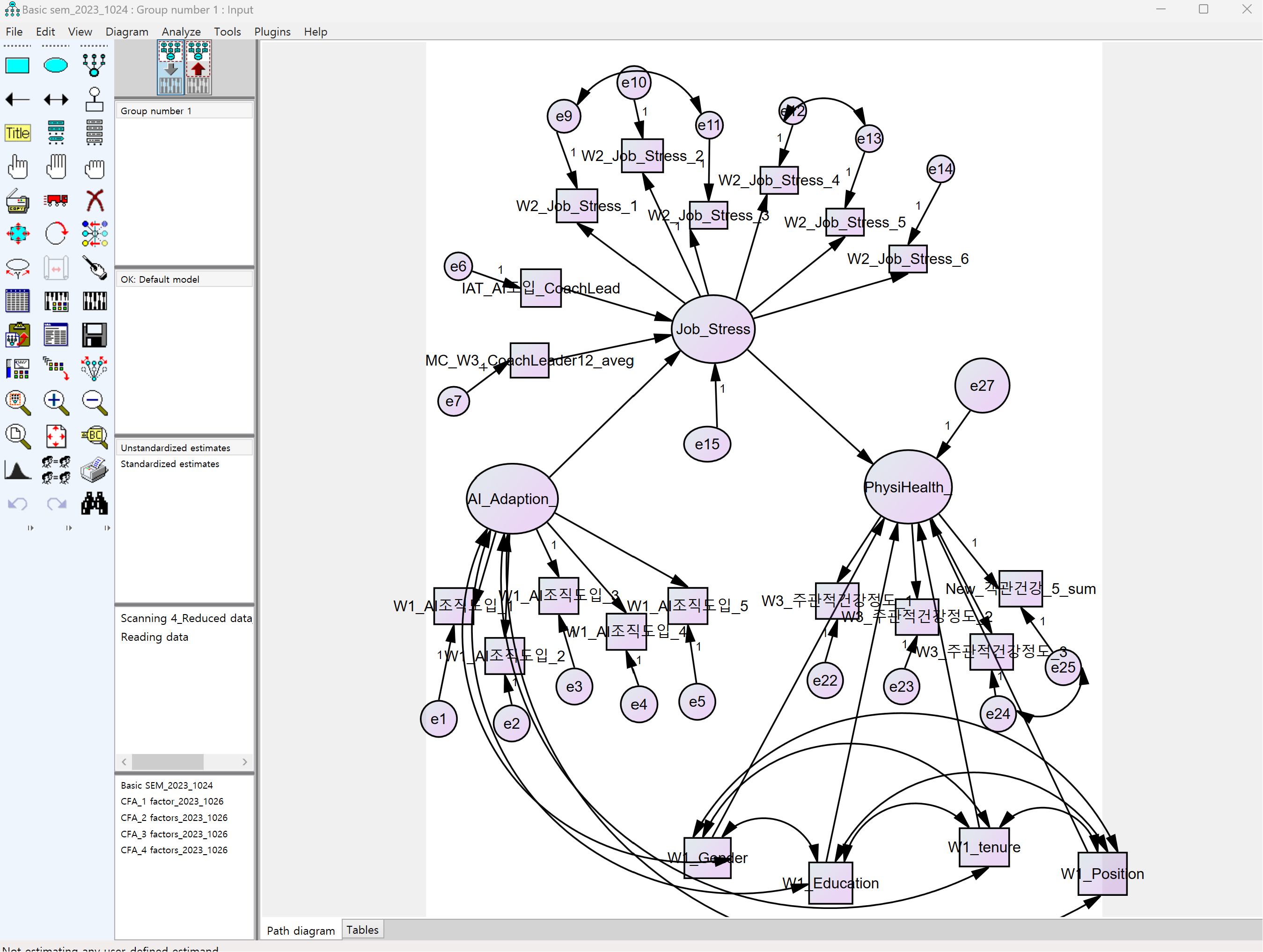

Supplement: Supplementary file 5 [file Image_5.PNG]
